# The impact of medication on osseointegration and implant anchorage in bone determined using removal torque—A review

**DOI:** 10.1016/j.heliyon.2022.e10844

**Published:** 2022-10-03

**Authors:** Martina Jolic, Sonali Sharma, Anders Palmquist, Furqan A. Shah

**Affiliations:** Department of Biomaterials, Sahlgrenska Academy, University of Gothenburg, Gothenburg, SE-405 30, Sweden

**Keywords:** Bone, Osseointegration, Implant, Removal torque, Drug delivery, Bisphosphonates, *In vivo*

## Abstract

Permanently anchored metal implants are frequently used in dental, craniomaxillofacial, and orthopaedic rehabilitation. The success of such therapies is owed to the phenomenon of osseointegration—the *direct* connection between the living bone and the implant. The extent of biomechanical anchorage (i.e., physical interlocking between the implant and bone) can be assessed with removal torque (RTQ) measurement. Implant anchorage is strongly influenced by underlying bone quality, involving physicochemical and biological properties such as composition and structural organisation of extracellular matrix, extent of micro-damage, and bone turnover. In this review, we evaluated the impact of various pharmacological agents on osseointegration, from animal experiments conducting RTQ measurements. In addition to substances whose antiresorptive and/or anti-catabolic effects on bone are well-documented (e.g., alendronate, zoledronate, ibandronate, raloxifene, human parathyroid hormone, odanacatib, and the sclerostin monoclonal antibody), positive effects on RTQ have been reported for substances that do not primarily target bone (e.g., aminoguanidine, insulin, losartan, simvastatin, bone morphogenetic protein, alpha-tocopherol, and the combination of silk fibroin powder and platelet-rich fibrin). On the contrary, several substances (e.g., prednisolone, cyclosporin A, cisplatin, and enamel matrix derivative) tend to adversely impact RTQ. While morphometric parameters such as bone-implant contact appear to influence the biomechanical anchorage, increased or decreased RTQ is not always accompanied by corresponding fluctuations in bone-implant contact. This further confirms that factors such as bone quality underpin biomechanical anchorage of metal implants. Several fundamental questions on drug metabolism and bioavailability, drug dosage, animal-to-human translation, and the consequences of treatment interruption remain yet unanswered.

## Highlights

• Effects of pharmacological agents on osseointegration of metal implants are reviewed.
• Removal torque is considered as an objective measure of osseointegration.
• Most investigated anabolic agents are alendronate, zoledronate, and ibandronate.
• hPTH has a positive effect while cyclosporin A and cisplatin have negative effects.
• Correction of underlying systemic disorders leads to improved osseointegration.

## Graphical abstract

**Figure:**
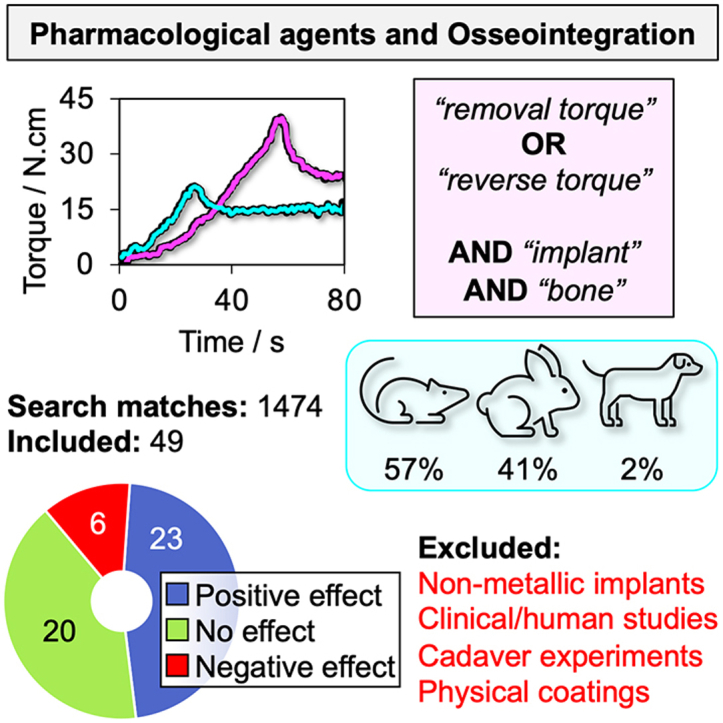

## 1 Introduction

Metal implants are widely used as part of dental, craniomaxillofacial, and orthopaedic rehabilitation. The longevity of such treatments depends on successful osseointegration—first described with reference to screw-shaped titanium implants as a *direct* connection between living bone and the implant, without interposed (soft) tissue (Adell et al., 1981). Later, this definition evolved to encompass a method for evaluating osseointegration (“*… on the light microscope level…*”) (Albrektsson et al., 1981), which not only enabled understanding the kinetics of peri-implant osteogenesis in terms of morphometric parameters such as bone-implant contact (BIC, %) and bone area (BA, %) (Sennerby et al., 1993), but also allowed for comparison between different bulk materials (Thomsen et al., 1997, Johansson and Albrektsson, 1991) and surface finishes (Larsson et al., 1997). It was soon discovered that functionally-loaded, human dental implants need not exhibit BIC up to 100% in order to remain clinically-stable (Sennerby et al., 1991)—i.e., the presence of (at least) some interposed tissue may be unavoidable and does not preclude long-term clinical success.

**Figure 1:**
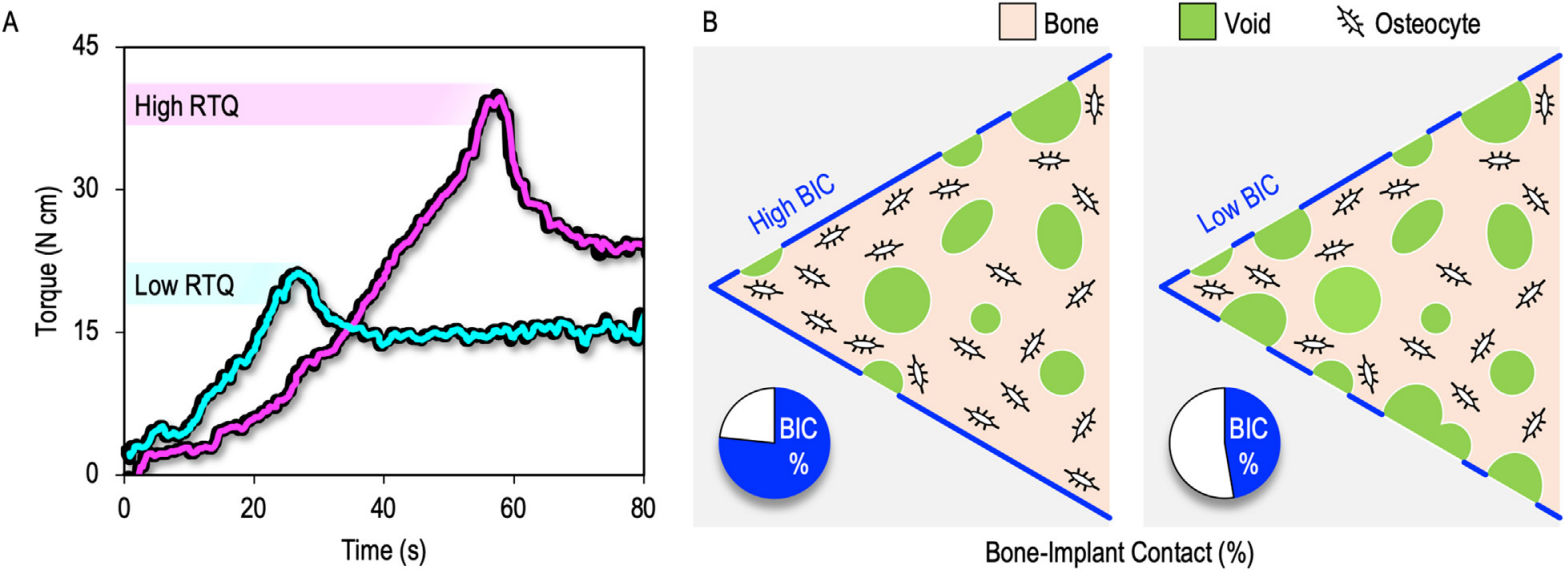
Removal torque (RTQ) and bone-implant contact (BIC). (A) Typical RTQ load-displacement curve. The peak/maximum torque value represents the point of failure (also referred to as the “break-point”) of the bone-implant interface. (B) BIC is one of several key factors contributing to the recorded RTQ.

Clinically, resonance frequency analysis (RFA) is a non-invasive method that is often used to assess implant stability as a function of stiffness/rigidity of the bone-implant system (Meredith et al., 1996). The reliability of RFA is, however, questionable due to many confounding factors that influence such measurements (e.g., implant geometry, bone type, gender, bone quality, etc.) (Huang et al., 2020, Haïat et al., 2014). Experimentally, measurement of removal torque (RTQ) is commonly used to assess biomechanical anchorage of implants in bone (Figure 1). While applying a continuous rotational force to the implant, the maximum force necessary to loosen the implant is recorded (Claes et al., 1976, Johansson and Albrektsson, 1987).

Although BIC may be viewed as a quantitative measure of osteoconduction (Albrektsson and Johansson, 2001), and correlation between BIC and RTQ values is reported (Shah et al., 2016), the distribution of nanoscale flaws (e.g., pores) within the extracellular matrix strongly influences the mechanical behaviour and damage resistance of bone (Tertuliano and Greer, 2016). Furthermore, local variations in Young's modulus of the extracellular matrix (e.g., between lamellar and interstitial bone or at the sub-lamellar level) (Gupta et al., 2006) and changes in extracellular matrix composition that lead to altered tissue-level mechanical competence (Morgan et al., 2018) imply that BIC, by itself, reveals an incomplete picture of osseointegration. Rather analogous to BIC, RTQ probes the extent of direct physical interlocking between the implant and living bone (Brånemark et al., 2011). Finite element modelling of the bone-implant interface also suggests that RTQ tends to be more reliable than pull-out tests (Rittel et al., 2018).

Implant anchorage is strongly influenced by the underlying bone quality (Palmquist, 2018), which is a broad term encompassing a wide range of chemical, physical, and biological characteristics such as extracellular matrix composition, structural organisation, extent of micro-damage, and bone turnover (Seeman and Delmas, 2006, Fratzl et al., 2004). Many of these parameters are only accessible *ex situ* (Hunt and Donnelly, 2016). Not only do various diseases affect bone material properties (Unnanuntana et al., 2011), a wide variety of drugs also alter bone metabolism (Goodman et al., 2007), particularly those used in the treatment of non-skeletal disorders (Watts, 2017). Here, we review the evidence from *in vivo* animal studies investigating the impact of pharmacological agents on osseointegration, where RTQ has been used for quantitative assessment of implant anchorage.

## 2 Methods

Using MEDLINE®/PubMed® and ScienceDirect, the following search term (or equivalent) was applied: (“**removal torque**” OR “**reverse torque**”) AND “**implant**” AND “**bone**”, which resulted in a total of 1474 matches. Duplicates and non-English language publications were discarded. Additional exclusion criteria were review papers, case reports, use of non-metallic implants, clinical/human studies, cadaver experiments, use of physical coatings (e.g., calcium phosphates), and *in vivo* studies that did not report RTQ data. Furthermore, four publications were excluded due to concerns over potential misrepresentation of experimental groups, and one publication was missing an appropriate ‘control/comparative’ group. Based on these criteria, 49 publications were included.

## 3 Review

The animal models used are rat (∼57%), rabbit (∼41%), and dog (∼2%). Various purpose-bred animal models of human disease have been used in addition to healthy animals (i.e., without underlying, experimentally-induced systemic disorders). These include animal models of diabetes mellitus, osteoporosis, and cardiovascular disease (Table 1). The typical administrative routes include subcutaneous and intravenous injections, oral (e.g., in drinking water, in the diet, or via gavage) administration, topical application at the implantation site, and local release (adsorbed or immobilised) from the implant surface. Implants are made out of commercially-pure titanium (cp-Ti), titanium-aluminium-vanadium alloy (Ti6Al4V), and stainless steel (SS). In a majority of cases, the therapeutic agent is administered for the entire duration of the experiment, starting from the time of implant insertion. In some cases, however, administration of therapeutic agents was initiated either before implant insertion (pre-insertion) or after implant insertion (post-insertion).

**Table 1:** Summary of the published literature.

Drug	Mode	Species	Experiment	Site	Healing period (days)	Δ RTQ (%)	Δ BIC (%)	Reference
Ibandronate	Local	Rat, SD	*±* Ibandronate∗∗	Tibia	14	60.9	−2.7	(Skoglund et al., 2004)
Ibandronate	Local	Rat, SD	Fibrinogen *±* Ibandronate∗∗	Tibia	14	127.3	-	(Wermelin et al., 2008)
Ibandronate	Local	Rat, Wistar	*±* Ibandronate	Tibia	14	19.7	-	(Lee et al., 2011)
					28	22.3	-	
Ibandronate	Systemic	Rat, SD	*±* Ibandronate	Femur	28	2.1	0.7	(Lotz et al., 2019)
			OVX *±* Ibandronate			10.3	8.7	
Alendronate	Local	Rat, SD	*±* Alendronate	Tibia	28	57.7	-	(Harmankaya et al., 2013)
Alendronate	Systemic	Rat, SD	OVX *±* Alendronate	Femur	28	57.8	21.5	(Narai and Nagahata, 2003)
Alendronate	Systemic	Rat, Wistar	OVX *±* Alendronate	Tibia	28	−14.5	82.2	(Shibamoto et al., 2018)
			OVX + WBV *±* Alendronate			−19.2	3.9	
Alendronate	Systemic	Rat, Wistar	*±* Alendronate	Tibia	5	18.6	18.4	(Verzola et al., 2015)
					10	91.5	−16.8	
					15	70.0	30.9	
					20	208.3	70.6	
					25	76.0	−7.9	
					30	78.6	20.4	
					45	−4.2	11.6	
					60	−4.3	29.7	
Alendronate	Systemic	Rat, Wistar	OVX *±* Alendronate	Tibia	150	72.8	-	(Giro et al., 2007)
Alendronate	Systemic	Rat, SD	DXM *±* Alendronate	Maxilla	14	−25.3	-	(Abtahi et al., 2013)
Alendronate	Systemic	Rat, Wistar	OVX *±* Alendronate	Tibia	42	110.0	17.1	(Ramalho-Ferreira et al., 2015)
Alendronate	Systemic	Rabbit, NZW	*±* Alendronate	Femur	28	0.9	-	(Chacon et al., 2006)
				Tibia	28	−10.6	-	
Alendronate	Local	Rabbit, NZW	*±* Alendronate	Tibia	28	−51.1	−60.2	(Guimarães et al., 2015)
Zoledronate	Local	Rat, SD	DXM *±* Zoledronate	Maxilla	14	64.6	-	(Abtahi et al., 2013)
Zoledronate	Local	Rat, Wistar	OVX *±* Zoledronate	Tibia	14	−2.3	−33.1	(Stadlinger et al., 2013)
					28	27.5	303.7	
Zoledronate	Local	Rat, Wistar	OVX *±* Zoledronate	Tibia	14	−1.2	−25.1	(Korn et al., 2019)
					28	27.7	304.7	
Zoledronate	Local	Rat, SD	OVX *±* Zoledronate	Femur	56	161.1	54.7	(Ma et al., 2017)
			OVX + Dopa *±* Zoledronate			8.2	13.9	
Zoledronate	Systemic	Rabbit, NZW	OVX *±* Zoledronate	Tibia	56	46.1	19.6	(Yildiz et al., 2010)
Zoledronate	Local	Rabbit, NZW	*±* Zoledronate	Femur	21	75.2	-	(Kwon et al., 2017)
Odanacatib	Systemic	Rat, SD	OVX *±* Odanacatib l.d.	Femur	56	33.6	0.0	(Yi et al., 2017)
			OVX ± Odanacatib h.d.			98.4	6.9	
SOSTab	Systemic	Rat, Wistar	OVX *±* SOSTab	Tibia	14	16.8	37.6	(Korn et al., 2019)
					28	7.9	125.7	
Zoledronate + SOSTab	Local + Systemic	Rat, Wistar	OVX + SOSTab *±* Zoledronate	Tibia	14	4.7	32.1	(Korn et al., 2019)
					28	102.8	440.1	
Raloxifene	Local	Rat, SD	*±* Raloxifene^†^	Tibia	28	66.7	-	(Harmankaya et al., 2013)
Raloxifene	Systemic	Rat, Wistar	OVX *±* Raloxifene	Tibia	42	323.0	302.2	(Ramalho-Ferreira et al., 2015)
hPTH	Systemic	Rat, SD	*±* hPTH∗∗	Tibia	28	218.2	-	(Skripitz and Aspenberg, 2001)
hPTH	Systemic	Rabbit, NZW	*±* hPTH	Tibia	28	28.1	-	(Corsini et al., 2008)
					56	21.8	-	
hPTH	Systemic	Rat, Wistar	OVX *±* hPTH	Tibia	28	24.7	143.2	(Shibamoto et al., 2018)
			OVX + WBV *±* hPTH			64.1	67.3	
17 β-estradiol	Systemic	Rat, Wistar	OVX *±* 17 β-estradiol	Tibia	150	42.8	-	(Giro et al., 2007)
Aminoguanidine	Systemic	Rat, Wistar	DM *±* Aminoguanidine l.d.	Femur	28	59.3	-	(Guimarães et al., 2011)
			DM *±* Aminoguanidine h.d.			115.3	-	
Insulin	Systemic	Rat, Wistar	DM *±* Insulin	Tibia	120	30.0	10.1	(de Molon et al., 2013)
Insulin	Systemic	Rabbit, NZW	DM *±* Insulin	Tibia	28	21.3	-	(Margonar et al., 2003)
					56	47.1	-	
					84	14.5	-	
Voglibose	Systemic	Rat, GK	DM *±* Voglibose	Tibia	21	34.1	−1.6	(Hashiguchi et al., 2014)
					63	11.1	35.7	
Losartan	Systemic	Rat, SHR or Wistar	*±* Losartan	Tibia	60	100.0	-	(Mulinari-Santos et al., 2018)
Simvastatin-Chitosan	Local	Rat, Wistar	OVX *±* Simvastatin-Chitosan	Tibia	14	4.9	61.4	(Stadlinger et al., 2013)
					28	6.5	139.3	
Simvastatin	Local	Rabbit, NZW	*±* Simvastatin	Tibia	28	11.8	-	(Faraco-Schwed et al., 2014)
					56	35.8	-	
BMP	Local	Dog, Foxhound	± BMP	Mandible	21	126.2	394.6	(Bessho et al., 1999)
					84	17.7	9.8	
rhBMP-2	Local	Rabbit, NZW	*±* rhBMP-2	Tibia	28	74.1	44.9	(Zhang et al., 2021)
					84	24.6	22.0	
Alpha-tocopherol	Systemic	Rat, Wistar	*±* Alpha-tocopherol∗∗	Tibia	29	34.6	-	(Savvidis et al., 2020)
SF-PRF	Local	Rabbit, NZW	± SF-PRF	Tibia	56	38.8	180.22	(Jang et al., 2010)
Prednisolone	Systemic	Rabbit, NZW	*±* Prednisolone	Tibia	90	36.9	-	(Fujimoto et al., 1998)
Cyclosporin A	Systemic	Rabbit, NZW	*±* Cyclosporin A	Tibia	28	38.0	75.0	(Sakakura et al., 2003)
					56	−30.8	−4.6	
					84	−37.5	−138.7	
Cyclosporin A	Systemic	Rabbit, NZW	*±* Cyclosporin A	Tibia	84	93.0	-	(Sakakura et al., 2006)
Cisplatin	Systemic	Rat, Wistar	*±* Cisplatin	Tibia	30	−342.3	−221.1	(Dantas et al., 2019)
					60	−89.0	−157.1	
EMD	Local	Rabbit, NZW	*±* EMD	Tibia	42	−70.0	−20.0	(Stenport and Johansson, 2003)
DXM	Systemic	Rat, SD	*±* DXM	Maxilla	14	−12.1	-	(Abtahi et al., 2013)
rhFGF-4	Local	Rabbit, NZW	*±* rhFGF-4	Tibia	42	16.1	50.0	(Stenport et al., 2003)
hGH	Systemic	Rabbit, NZW	*±* hGH	Tibia	56	−5.3	−6.9	(Stenport et al., 2001)
Oxytocin	Systemic	Rabbit, NZW	*±* Oxytocin	Femur	31	−7.1	47.4	(Cho et al., 2014)
Nicotine tartrate	Systemic	Rabbit, NZW	*±* Nicotine tartrate	Tibia	14	−3.0	14.6	(Balatsouka et al., 2005b)
					28	−5.1	−1.8	
Nicotine tartrate	Systemic	Rabbit, NZW	*±* Nicotine tartrate	Tibia	14	−13.1	5.7	(Gotfredsen et al., 2009)
					28	1.7	−17.5	
Nicotine tartrate	Systemic	Rabbit, NZW	*±* Nicotine tartrate	Tibia	14	−10.6	4.5	(Balatsouka et al., 2005a)
					28	−14.6	−12.3	
Omeprazole	Systemic	Rat, SD	± Omeprazole l.d.	Tibia	28	−33.8	-	(Tekin et al., 2021a)
			± Omeprazole h.d.			21.3	-	
Propranolol	Systemic	Rat, SD	± Propranolol l.d.	Tibia	28	−32.3	-	(Tekin et al., 2021b)
			± Propranolol h.d.			0.0	-	
Solcoseryl	Systemic	Rabbit, JW	CCEF *±* solcoseryl	Femur	14	5.6	30.0	(Ochi et al., 2003)
Collagen-CS	Local	Rat, Wistar	OVX *±* Collagen-CS	Tibia	14	22.6	30.7	(Stadlinger et al., 2013)
					28	21.4	39.3	
Fibrinogen	Local	Rat, SD	*±* Fibrinogen	Tibia	14	−52.3	-	(Wermelin et al., 2008)
*S. officinale*	Systemic	Rat, Holtzman	*± S. officinale*	Tibia	7	18.6	-	(Spin-Neto et al., 2010)
					14	33.3	-	
					28	−12.1	-	
					56	−22.2	-	
ASU	Systemic	Rat, Wistar	*±* ASU	Tibia	60	6.0	24.0	(de Oliveira et al., 2014)
ASU	Systemic	Rat, Holtzman	Arthritis *±* ASU	Tibia	14	−20.4	14.6	(de Paula et al., 2018)
					30	41.4	37.0	
					60	2.1	50.9	
Caffeine	Systemic	Rat, Wistar	*±* Caffeine	Tibia	84	88.7	-	(Omar et al., 2021)

∗∗ = Stainless steel implants. † = Implant surface methylated with dichloromethylsilane prior to raloxifene adsorption. ASU = Avocado-Soybean unsaponifiables. BMP = Bone morphogenetic protein. CS = Chondroitin sulphate. DM = Diabetes mellitus. Dopa = 3,4-dihydroxy-L-phenylalanine. DXM = Dexamethasone. EMD = Enamel matrix derivative. GK = Goto-Kakizaki. h.d. = High dose. hGH = Human growth hormone. hPTH = Human parathyroid hormone. JW = Japanese white. l.d. = Low dose. NZW = New Zealand white. OVX = Ovariectomised. rhBMP-2 = Recombinant human bone morphogenetic protein-2. rhFGF-4 = Recombinant human fibroblast growth factor-4. SD = Sprague Dawley. SF-PRF = Silk fibroin powder and platelet-rich fibrin. SHR = Spontaneously hypertensive rats. SOSTab = Sclerostin monoclonal antibody. WBV = Whole-body vibration. Δ BIC = % difference between mean BIC of groups with (test) and without (control) therapeutic agent. Δ RTQ = % difference between mean RTQ of groups with (test) and without (control) therapeutic agent.

## 4 Substances with anabolic and/or anti-catabolic effects on bone

**Figure 2:**
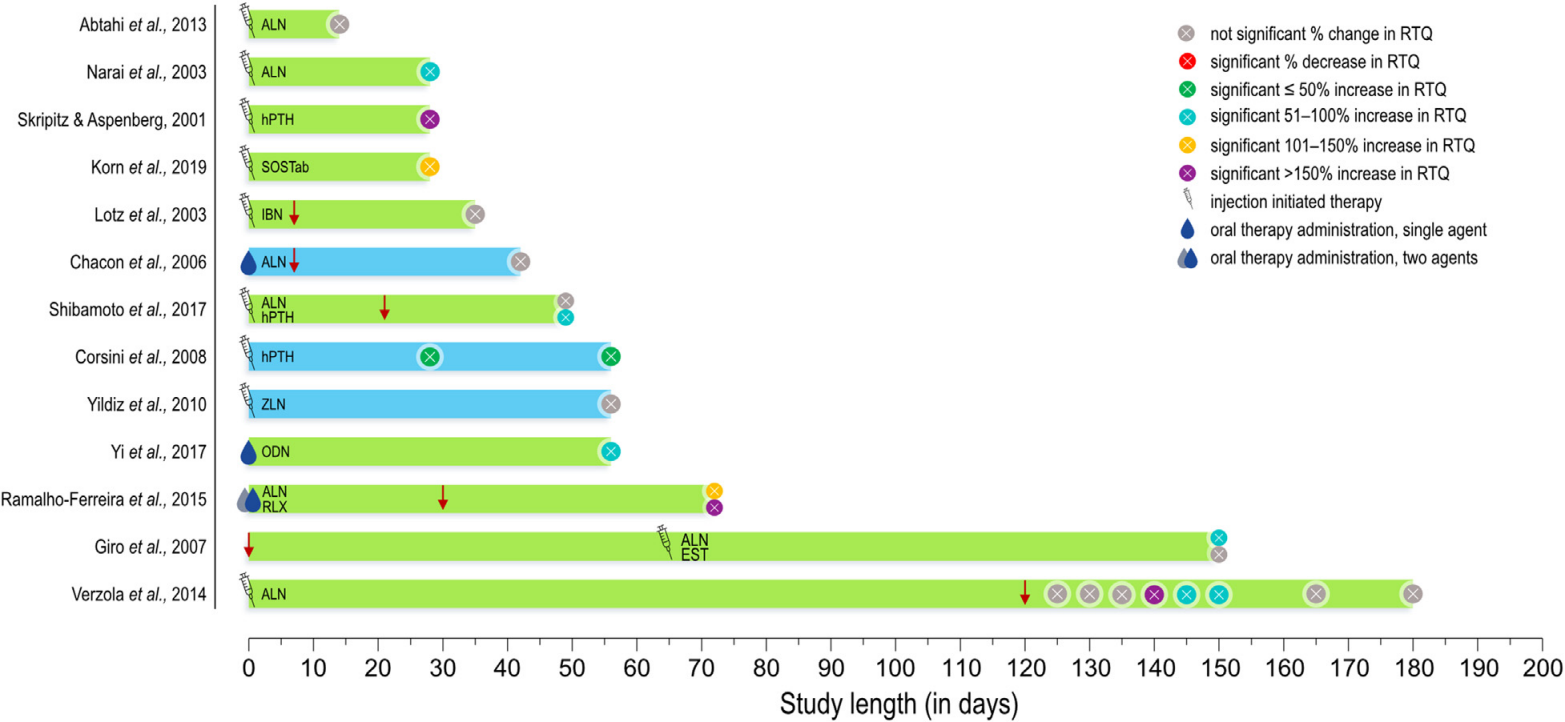
Systemic administration of antiresorptive agents. Green bars: *Rattus norvegicus* (rat) models. Blue bars: *Oryctolagus cuniculus* (rabbit) models. Down arrow: implant placement when different to initiation of therapy. Circle: study endpoint(s). ALN ​= ​Alendronate. hPTH ​= ​Human parathyroid hormone. SOSTab ​= ​Sclerostin monoclonal antibody. IBN ​= ​Ibandronate. ZLN ​= ​Zoledronate. ODN ​= ​Odanacatib. RLX ​= ​Raloxifene. EST ​= ​17 β-estradiol.

**Figure 3:**
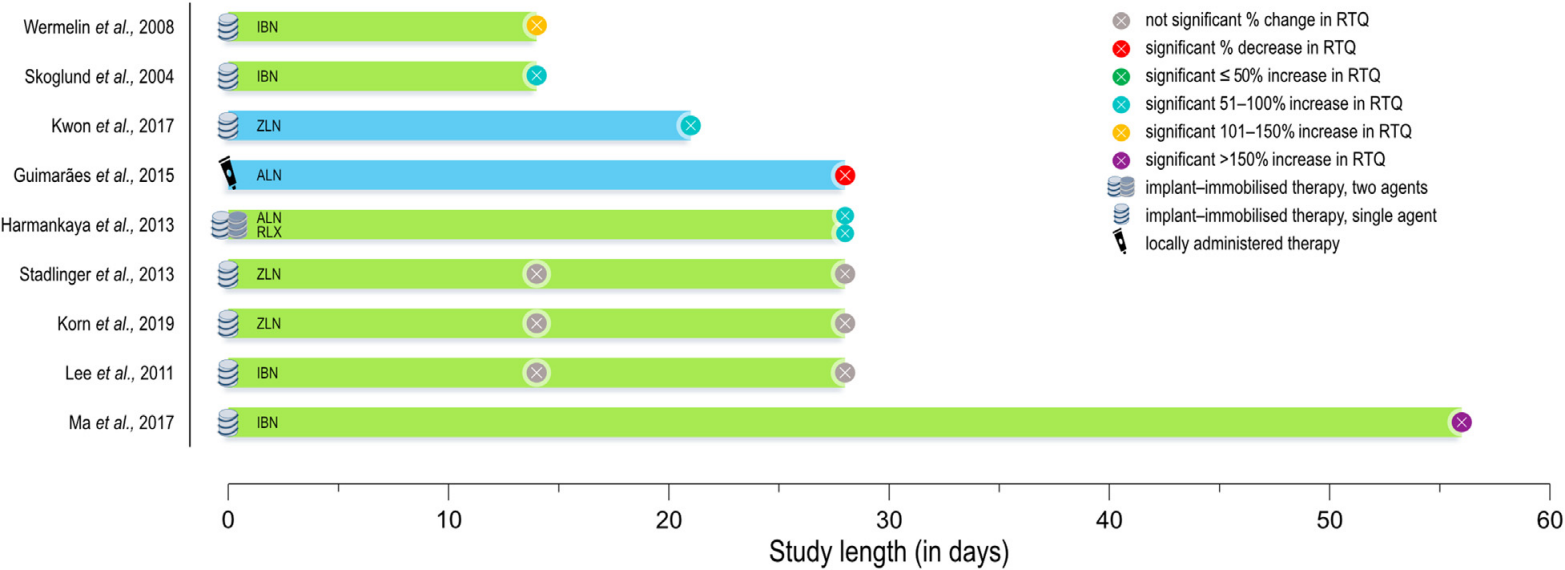
Local administration of antiresorptive agents. Green bars: *Rattus norvegicus* (rat) models. Blue bars: *Oryctolagus cuniculus* (rabbit) models. Circle: study endpoint(s). IBN ​= ​Ibandronate. ZLN ​= ​Zoledronate. ALN ​= ​Alendronate. RLX ​= ​Raloxifene.

Four bisphosphonates (BPs), ibandronate (IBN), alendronate (ALN), zoledronate (ZLN), and pamidronate as well as hormone therapies, cathepsin-K inhibitor (odanacatib), and sclerostin monoclonal antibody have been administered via systemic (Figure 2) and local (Figure 3) routes. Studies were performed in animals with experimentally-induced osteoporosis, e.g., by ovariectomy, and/or in healthy animals.

### 4.1 Ibandronate

Local injection of IBN to the implantation site immediately prior to implant placement, significantly increases the mechanical anchorage of SS implants in rats. Compared to saline-treated sites, 60% higher RTQ at 2 weeks post-insertion indicates that the expected effects of locally administered IBN are detectable during early stages of healing. The BIC, however, remains unchanged (Skoglund et al., 2004). Similarly, SS implants with covalently bound fibrinogen and pamidronate, and incubated overnight in a solution of IBN, show higher RTQ values at 2 weeks of healing in the rat tibia (Wermelin et al., 2008). In contrast, local release of IBN from anodically oxidised titanium implants has not been found to be a successful strategy, where only a ‘trend’ in RTQ increase (without being statistically significant) has been noted at 2- and 4 weeks of healing in rats (Lee et al., 2011). Evaluation of systemic IBN administration on osseointegration was carried out in ovariectomised (OVX) rats. Here, subcutaneous IBN injections, once before and once after implant placement, showed no effect on biomechanical stability or the BIC. The effect(s) of IBN were isolated by incorporating pair feeding and a phytoestrogen-free diet into the experimental setup (Lotz et al., 2019).

### 4.2 Alendronate

Mesoporous TiO_2_ films deposited on screw-shaped implants offer a relatively simple but promising strategy for targetted, controlled release of ALN, where significantly increased RTQ has been reported at 4 weeks of healing in the rat tibia (Harmankaya et al., 2013). In OVX rats, systemic administration of ALN has been shown to result in increased RTQ at 4 weeks of healing. Here, OVX rats treated with ALN were able to attain RTQ values comparable to those of non-OVX rats without ALN administration (Narai and Nagahata, 2003). Systemic ALN administration has also been investigated in combination with whole-body vibration as a form of low-magnitude, high-frequency loading. Starting 3 weeks prior to implant placement, ALN treatment failed to induce significant improvement in RTQ, either alone or in combination with whole-body vibration (Shibamoto et al., 2018). The consequences of long-term ALN use have been simulated in rats by weekly subcutaneous ALN injections for 120 days prior to implantation. Here, higher RTQ was recorded at 20-, 25-, and 30 days post-insertion while increases in BIC were only observed at 20- and 60 days (Verzola et al., 2015). Likewise, a positive impact of ALN has been observed when peri-implant healing (i.e., osseointegration) has occurred prior to the onset of osteoporosis in rats. Beginning at 5 days following an OVX, subcutaneous ALN administration every other day led to a higher RTQ of tibial implants at 90 days post-OVX. Moreover, the increase in RTQ was associated with higher bone mineral density of the femur and the lumbar vertebrae measured using dual-energy X-ray absorptiometry (Giro et al., 2007).

The use of BPs has been linked to the occurrence of the osteonecrosis of the jaw in the vicinity of dental implants (Martins et al., 2021). Experimentally, osteonecrosis can be achieved with the combined use of dexamethasone and a bisphosphonate (Abtahi et al., 2012). Administration of dexamethasone, once before and once after implant insertion, together with daily subcutaneous ALN injections leads to osteonecrosis in peri-implant areas. While development of osteonecrosis does not translate into decreased RTQ, systemic administration of ALN also does not restore the bone formation–resorption balance since comparatively lower bone volume appears to persist (Abtahi et al., 2013). In OVX rats, higher RTQ values are also reported at 6 weeks of healing under systemic administration of ALN by oral gavage. Here, no differences have been noted in relation to topological modification, i.e., machined surface vs. acid etched surface (Ramalho-Ferreira et al., 2015).

Weekly oral administration of ALN via an oropharyngeal tube has not been shown to affect RTQ values in rabbits after five weeks of healing. Higher RTQ values are reported for bicortical insertion (i.e., in the tibia) compared to monocortical insertion (i.e., in the femur) (Chacon et al., 2006). In the same animal model, topical application of a high concentration sodium alendronate gel, containing 10 mg ALN, directly at the implantation site immediately before implant placement, caused a ∼50% decrease in RTQ, together with absence of neo-bone formation and presence of granular tissue within the implant threads (Guimarães et al., 2015).

### 4.3 Zoledronate

ZLN is a highly potent, third-generation BP, used in the treatment of osteoporosis, Paget's disease, and bone-related adverse effects of myeloma and cancers (Reid et al., 2020). In healthy (i.e., non-osteoporotic) rats, the use of a thin fibrinogen coating as a carrier for local delivery of ZLN from screw-shaped implants significantly increases RTQ values in the maxilla. Additional administration of dexamethasone, once before and once after implant insertion, does not lead to the development of osteonecrosis (Abtahi et al., 2013). In OVX rats, spray-coating ZLN on titanium screws, despite a significant increase in BIC at 4 weeks of healing, is ineffective at increasing RTQ at 2- or 4 weeks of healing (Korn et al., 2019, Stadlinger et al., 2013). At 8 weeks of healing in OVX rats, a demonstrable effect of adsorbed ZLN has been reported (Ma et al., 2017), but simultaneous release of ZLN and 3,4-dihydroxy-L-phenylalanine from the implant surface does not have apparent synergistic effects on RTQ (Ma et al., 2017).

In OVX rabbits, a single intravenous dose of ZLN prior to implant insertion does not improve biomechanical properties of osseointegrated implants after 8 weeks of healing, despite statistically significant increases in both RFA and BIC (Yildiz et al., 2010). In the rabbit femur, higher RTQ at 3 weeks of healing has been reported in association with local release of ZLN from TiO_2_ nanotubes (TiNT), with similar trends in implant stability measured using RFA, but no changes were apparent in new bone formation within the implant threads (Kwon et al., 2017).

### 4.4 Odanacatib and sclerostin monoclonal antibody

In view of the known adverse effects of BPs, their use in implant healing and long-term maintenance of peri-implant bone remains debated and highlights the need for alternative solutions (Stavropoulos et al., 2018). One of the proposed alternatives to BP therapy is odanacatib, a highly selective and sensitive inhibitor of cathepsin K, commonly used in the treatment of postmenopausal osteoporosis (Mcclung et al., 2019). In OVX rats, a dose-dependent effect of daily, oral administration of odanacatib on RTQ has been reported (Yi et al., 2017). Odanacatib has since been discontinued by Merck & Co. due to an increased risk of cardiovascular events such as stroke (Mullard, 2016). Another alternative to BPs is the sclerostin monoclonal antibody (SOSTab), which appears to have no effect on RTQ at either 2- or 4 weeks of healing in OVX rats (Korn et al., 2019). However, synergistic effects have been reported for systemic administration of SOSTab in combination with spray-coated ZLN at 4 weeks of healing (Korn et al., 2019).

### 4.5 Hormones and hormone modulators

Hormones such as human parathyroid hormone (hPTH), 17 β-estradiol, and oestrogen receptor moderator raloxifene (RLX) have also been investigated for their potential effects on implant anchorage. Locally released RLX from a mesoporous TiO_2_ film, significantly improves RTQ after 4 weeks of healing (Harmankaya et al., 2013). Similarly, higher RTQ values are reported in OVX rats at 6 weeks of healing where RLX is administered by oral gavage (Ramalho-Ferreira et al., 2015). In rats, hPTH administered daily for 4 weeks trebles the mechanical anchorage of screw-shaped SS implants (Skripitz and Aspenberg, 2001). Moreover, in OVX rats, significantly higher RTQ has been observed at 4 weeks of healing due to the combined effect of subcutaneous hPTH administration, starting 3 weeks before implant insertion, and whole-body vibration (Shibamoto et al., 2018). The positive effect of hPTH, administered 3 times per week, has also been demonstrated in rabbits at short (e.g., 4 weeks) and intermediate (e.g., 8 weeks) healing periods (Corsini et al., 2008). The use of 17 β-estradiol has been investigated in rats where the onset of osteoporosis (by ovariectomy) occurs after initial peri-implant healing has been allowed to take place. However, beginning 5 days post-OVX, daily subcutaneous administration of 17 β-estradiol has not been demonstrated to have a significant impact on RTQ at 150 days of healing (Giro et al., 2007).

## 5 Substances without primary effects on bone

**Figure 4:**
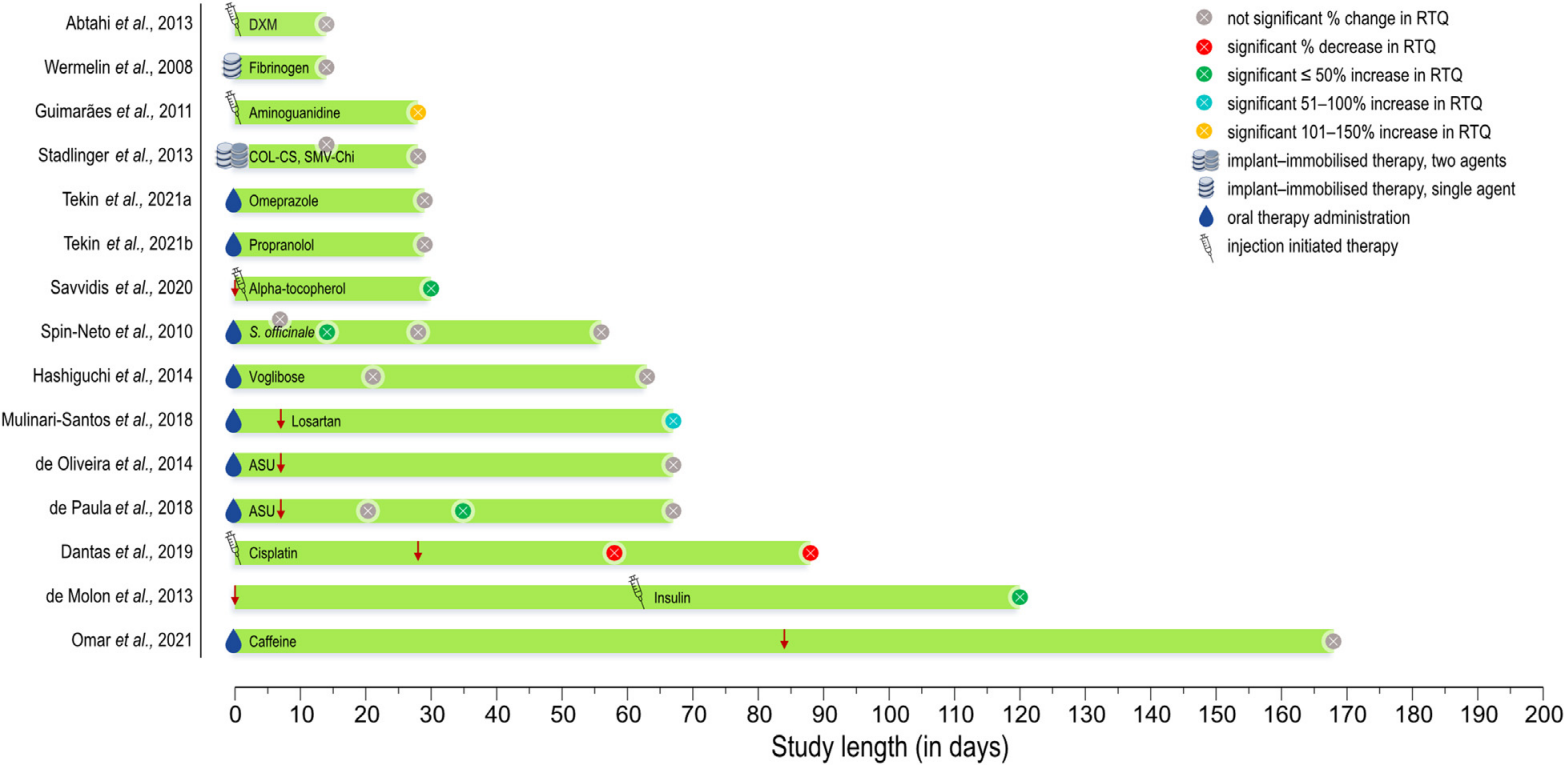
Studies in *Rattus norvegicus* (rat) models. Down arrow: implant placement when different to initiation of therapy. Circle: study endpoint(s). COL-CS = Collagen-Chondroitin sulphate. SMV-Chi = Simvastatin-Chitosan. ASU = Avocado-Soybean unsaponifiables.

**Figure 5:**
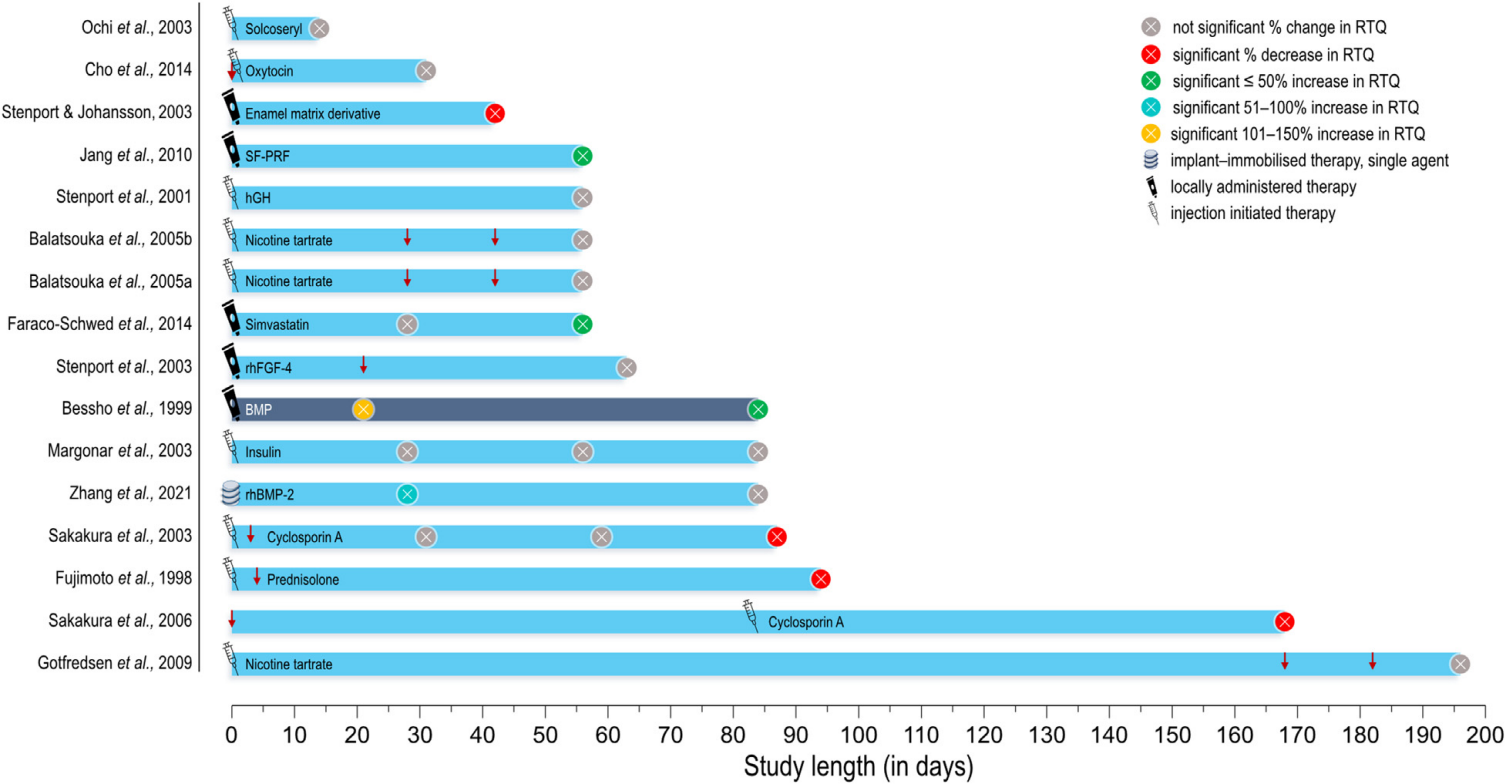
Studies in *Oryctolagus cuniculus* (rabbit) and *Canis lupus familiaris* (dog) models. Down arrow: implant placement when different to initiation of therapy. Circle: study endpoint(s). SF-PRF = Silk fibroin powder and platelet-rich fibrin. hGH = Human growth hormone. rhFGF-4 = Recombinant human fibroblast growth factor-4. BMP = Bone morphogenetic protein. rhBMP-2 = Recombinant human bone morphogenetic protein-2.

Various other substances that are either commonly used for the treatment of different systemic disorders or indirectly influence bone quality have been evaluated for their impact on osseointegration. Similar to antiresorptives and anti-catabolic drugs, these have been administered both systemically and locally in rat (Figure 4), rabbit, and dog (Figure 5) models.

### 5.1 Substances with positive effects on RTQ

#### 5.1.1 Aminoguanidine

The effect(s) of hyperglycaemia on osseointegration have been investigated in various rodent models of diabetes mellitus (King et al., 2016). Here, the animals are either genetically modified to develop the disease by breeding or gene targeting, or have the disease chemically induced (King et al., 2016). Insulin-dependent diabetes mellitus can be induced by intravenous administration of alloxan monohydrate (Shaw Dunn and Mcletchie, 1943). A dose-dependent (10 mg/kg vs. 20 mg/kg), positive effect of aminoguanidine has been reported in alloxan monohydrate induced diabetes mellitus. RTQ values in untreated hyperglycaemic rats remain lower than in normoglycaemic (i.e., healthy) rats. Daily administration of aminoguanidine significantly improves RTQ in hyperglycaemic rats. On the contrary, normoglycaemic animals are unresponsive to similar doses of aminoguanidine (Guimarães et al., 2011).

#### 5.1.2 Insulin

In rats where diabetes mellitus is induced with a single dose of streptozotocin, hyperglycaemia leads to lower RTQ than in the normoglycaemic state. However, systemic administration of insulin significantly improves the biomechanical anchorage, where RTQ at 16 weeks of healing is comparable to that in normoglycaemic animals (de Molon et al., 2013). However, in rabbits with alloxan monohydrate induced diabetes mellitus, systemic administration of insulin does not lead to a significant improvement in RTQ values compared to hyperglycaemic animals. In contrast to hyperglycaemic states (treated or untreated), a progressive improvement in RTQ is reported in normoglycaemic animals at 8- and 12 weeks of healing (Margonar et al., 2003).

#### 5.1.3 Losartan

Losartan, an angiotensin II receptor antagonist, positively influences osseointegration in spontaneously hypertensive rats (Mulinari-Santos et al., 2018). Administration of losartan via drinking water (30 mg/kg per day), starting one week prior to implant placement until 60 days of healing, leads to an increase in RTQ together with improvement in bone microstructure, e.g., bone volume and trabecular thickness. Importantly, the same pattern is not evident in normotensive Wistar rats (Mulinari-Santos et al., 2018).

#### 5.1.4 Simvastatin

Simvastatin is a hypolipidemic agent. In OVX rats, simvastatin adsorbed on titanium screws (∼35 μg per implant) does not show a considerable effect on RTQ at either 2- or 4 weeks of healing (Stadlinger et al., 2013). In contrast, a positive effect of topical application of simvastatin (7.5 mg) directly at the implantation site immediately before implant insertion has been shown in rabbits, where the RTQ is ∼36% higher at 8 weeks of healing (Faraco-Schwed et al., 2014). Interestingly, application of simvastatin also led to a significant improvement in RTQ between 4- and 8 weeks of healing, which was not observed in the absence of simvastatin. The mechanism of action, here, remains unclear and it cannot be accurately discerned whether simvastatin accelerates the early stages of healing or delays remodelling, or otherwise alters bone material properties on the extracellular matrix level.

#### 5.1.5 Bone morphogenetic protein

A promising outcome is reported for the combination of bovine-derived bone morphogenetic protein (BMP) and atelocollagen (1:600 ratio) injected directly at the implantation site prior to implant insertion. Significant improvement in RTQ values is noted at both 3- and 12 weeks of healing in dogs. Not unexpectedly, the difference in RTQ is more pronounced at 3 weeks than at 12 weeks (Bessho et al., 1999). The combination of TiNT and poly(lactic-*co*-glycolic acid) has been exploited as a release system for local delivery of recombinant human bone morphogenetic protein-2 (rhBMP-2) (Zhang et al., 2021). Here, poly(lactic-*co*-glycolic acid) coating thickness governs the release kinetics and therefore must be tailored to prevent a rapid *burst* release and provide controlled, sustained discharge in order to enable bone regeneration. Compared to uncoated implants, a significant improvement in RTQ was noted at 4 weeks of healing in rabbits using the TiNT–poly(lactic-*co*-glycolic acid) system. However, differences were not observed at 12 weeks of healing (Zhang et al., 2021).

#### 5.1.6 Alpha-tocopherol

Reducing oxidative stress injury, associated with the production of reactive oxygen species, is yet another potential strategy for enhancing osseointegration. An example is the use of the vitamin E isomer alpha-tocopherol. When administered intraperitoneally in rats (40 mg/kg per day), starting at implant insertion, a small increase in the RTQ of screw-shaped SS implants is reported at 4 weeks of healing (Savvidis et al., 2020).

#### 5.1.7 Silk fibroin powder and platelet-rich fibrin

The combination of silk fibroin powder and platelet-rich fibrin has been found to be effective in improving implant anchorage. An increase of ∼39% in RTQ has been reported after 8 weeks of healing in rabbits, together with ∼180% increase in BIC (Jang et al., 2010).

### 5.2 Substances with negative effects on RTQ

#### 5.2.1 Prednisolone

Certain substances may, in fact, have adverse effects on osseointegration and the biomechanical anchorage of implants. The unfavourable consequences of corticosteroids such as prednisolone are reflected in lowered RTQ of implants placed in rabbit tibiae at 12 weeks of healing. At the same time, the three courses of prednisolone given for four days once a month, do not have a pronounced effect in the mandible, where only a minor reduction in RTQ values is noted (Fujimoto et al., 1998).

#### 5.2.2 Cyclosporin A

The immunosuppressive agent cyclosporin A leads to detrimental effects on RTQ in rabbits. Cyclosporin A administration beginning 3 days prior to implant insertion results in ∼27% decreased RTQ together with 58% lower BIC at 12 weeks of healing (Sakakura et al., 2003). Not only does cyclosporin A interfere with ongoing implant healing, it has been demonstrated that cyclosporin A administration for 12 weeks negatively impacts the mechanical anchorage of implants that have previously healed undisturbed for 12 weeks, where a ∼48% decrease in RTQ has been observed (Sakakura et al., 2006).

#### 5.2.3 Cisplatin

The antineoplastic chemotherapeutic drug cisplatin, administered intraperitoneally four times at one-week intervals prior to implant insertion, appears to have a profound negative impact on RTQ (Dantas et al., 2019). RTQ values were significantly lower, both at 30- and 60 days of healing in rats treated with cisplatin. However, bone morphological parameters such as BIC and RTQ show a gradual recovery with healing time, and without any alterations in Ca/P ratios of bone, suggesting that cisplatin primarily delays the reparative process.

#### 5.2.4 Enamel matrix derivative

Enamel matrix derivative, constituting up to 90% amelogenin, appears to have detrimental effects on RTQ at 6 weeks of healing in the rabbit tibia, when placed directly at the implantation site prior to implant insertion (Stenport and Johansson, 2003). While a significant reduction in BIC is not demonstrated, the reported decrease in total bone length points towards differences in the mechanical anchorage afforded by bone present at non-threaded areas (e.g., the apex) of screw-shaped implants. A likely explanation is that enamel matrix derivative primarily stimulates the regeneration of tissues such as the periodontal ligament and acellular cementum (Hammarström et al., 1997), whereas bone formation is attributable to functional adaptation.

### 5.3 Substances with no recorded effect on RTQ

#### 5.3.1 Voglibose

The Goto-Kakizaki rat maintained on a high fat and high glucose diet remains unresponsive to the alpha-glucosidase inhibitor drug voglibose, where no improvement in RTQ values has been recorded at either 3- or 9 weeks of healing. Nevertheless, RTQ values increase temporally, between 3- and 9 weeks, for both voglibose treated and untreated rats (Hashiguchi et al., 2014).

#### 5.3.2 Dexamethasone

Administration of dexamethasone, once before and once after implantation, leads to no detectable change in RTQ after 14 days of healing in rats. However, signs of osteonecrosis have been noted in a small proportion (1/10) of animals (Abtahi et al., 2013).

#### 5.3.3 Fibroblast growth factor-4, human growth hormone, and oxytocin

A single dose of amino-terminally truncated recombinant human fibroblast growth factor-4 (rhFGF-4), administered locally at the site of implant placement three weeks prior to implant insertion, does not result in a noteworthy improvement in RTQ at 6 weeks of healing in rabbits (Stenport et al., 2003). Here, the perceived inefficacy of rhFGF-4 is multifactorial. For instance, it remains unknown whether rhFGF-4 is present for long enough at a high enough concentration, or if any local improvement in bone quality is lost due to implant site preparation or upon eventual remodelling. In rabbits, a daily dose of human growth hormone (hGH) administered subcutaneously via a micro-osmotic pump for 8 weeks has also not led to a significant improvement in RTQ (Stenport et al., 2001). Similarly, intermittent administration of oxytocin twice per week fails to induce significant improvement in RTQ at 4 weeks of healing in rabbits (Cho et al., 2014).

#### 5.3.4 Nicotine tartrate

With smoking being a major global health concern (Pierce et al., 2011), the impact of systemically administered nicotine tartrate on osseointegration remains inconclusive. Several studies have failed to demonstrate a negative effect of combined pre-implantation and post-implantation nicotine tartrate exposure at doses of 30- and 60 mg/kg per week on either RTQ or BIC in rabbits (Balatsouka et al., 2005b, Gotfredsen et al., 2009, Balatsouka et al., 2005a). Whether it is long-term pre-implantation exposure (∼24–26 weeks) at the higher dose (Gotfredsen et al., 2009), or short-term pre-implantation exposure (∼4–6 weeks) (Balatsouka et al., 2005a, Balatsouka et al., 2005b), RTQ tends to increase with healing time independently of nicotine tartrate exposure parameters. Currently there are no known studies to confirm or reject the impact of nicotine tartrate on RTQ and bone material properties at extended healing/post-implantation periods.

#### 5.3.5 Omeprazole

Omeprazole, a proton pump inhibitor, administered at 72 h intervals via an orogastric tube (5–10 mg/kg) has not been found to influence RTQ at 4 weeks of healing in rats (Tekin et al., 2021a).

#### 5.3.6 Propranolol

Propranolol, an antihypertensive agent, administered thrice a week by oral gavage (5–10 mg/kg) has not been shown to affect RTQ at 4 weeks of healing in rats (Tekin et al., 2021b).

#### 5.3.7 Solcoseryl

The haemodialysate solcoseryl is a tissue respiration stimulating agent that has found some interest in the pursuit of improving osseointegration. When applied to deep wounds involving skin, muscle, and periosteum, solcoseryl tends to stimulate healing initially by provoking a strong inflammatory response and formation of granulation tissue, however, the inflammation is protracted and subsequent healing steps are delayed (Wilmink et al., 2000). The use of solcoseryl has been explored in combination with 60 kHz capacitively coupled electric field stimulation of Ti6Al4V implants in rabbits. While electrical field stimulation for a few hours per day appears to improve RTQ at 14 days, solcoseryl administration appears to have no synergistic effects on RTQ (Ochi et al., 2003).

#### 5.3.8 Natural-derived biopolymers

Reportedly, the combination of type I collagen (1 mg/mL) and chondroitin sulphate (50 μg/mL) as a coating on titanium implants has little or no impact on RTQ at either 2- or 4 weeks of healing in OVX rats (Stadlinger et al., 2013). Addition of ten layers of human plasminogen-free fibrinogen tends to lower the RTQ at 2 weeks of healing in rats (Wermelin et al., 2008). Such a negative effect has been discussed in the context of implant topography being masked by the fibrinogen rather than inhibition of biological processes that lead to the establishment of a direct bone-implant interface.

#### 5.3.9 Plant-derived substances

Taking inspiration from alternative medicine, derivatives of the plant *Symphytum officinale*, said to contain various substances including ∼0.8% allantoin, have been explored for enhancing osseointegration (Spin-Neto et al., 2010). Daily administration of *S. officinale* via drinking water can lead to a significant increase in RTQ at 2 weeks of healing in rats together with higher radiographic bone density. Subsequent increases in radiographic bone density (i.e., at 4- and 8 weeks of healing) are not associated with corresponding changes in RTQ, most likely due to continued bone apposition at some distance from the immediate bone-implant interface and therefore not contributing to resistance against torsion. Another plant-derived substance that has recently gained attention is the avocado-soybean unsaponifiables extract (ASU)—also known as piascledine. One study reports a lack of improvement in RTQ at 60 days of healing in the rat tibia in association with daily ASU administration (600 mg/kg), irrespective of whether ASU administration is started on the same day as implant insertion or 7 days earlier (de Oliveira et al., 2014). Under experimentally-induced arthritis in rats, and in contrast to otherwise healthy animals, starting ASU administration 7 days before implant insertion provokes ∼41% increase in RTQ at 30 days of healing. However, no significant effect is noted at 15- or 60 days of healing (de Paula et al., 2018). Not surprisingly, ASU is considered to have no quantifiable impact on joint space width deterioration in hip osteoarthritis (Maheu et al., 2014).

Beneficial effects of habitual caffeine intake on osseointegration have also been reported. In rats receiving 300 mg/L caffeine daily for 12 weeks prior to implant insertion, RTQ is ∼87% higher at 12 weeks of healing (i.e., 24 weeks of caffeine administration) (Omar et al., 2021). Higher RTQ is associated with higher bone area within the implant threads. It is unclear, however, if caffeine intake plays a direct pro-osteogenic role or if such effects are attributable to greater stimulation and physical activity, leading to increased physiological loading of the skeleton.

## 6 Discussion

From bisphosphonates to prednisolone (a corticosteroid), cyclosporin A (an immunosuppressive agent), and cisplatin (an antineoplastic drug), many therapeutic agents, either positively or negatively, influence the biomechanical anchorage of metal implants in bone. We examine the impact of various pharmacological substances on osseointegration using RTQ measurements as an objective measure of the amount of work required to disrupt the integrity of the bone-implant interface (i.e., 1 N cm = 0.01 J), thereby leading to implant loosening. When performed experimentally, such loosening is aseptic, though varying levels of trauma may be experienced by the surrounding tissues. Alternatively, pull-out and push-out tests are used to assess the biomechanical anchorage of implants, however, implant misalignment, implant migration during healing, and unstable crack propagation limit the reliability of these measurements (Gao et al., 2019). Such tests should be representative of the BIC in “flat” implants, but for screw-shaped implants, the total amount of bone within the implant thread could contribute to the forces measured—i.e., in that case, the pull-out test is significantly less restricted to the ‘bone-implant interface’.

For decades, histological assessment (by means of optical microscopy) has been the gold standard for bone morphometry (Revell, 1983, Parfitt et al., 1987), but more recently X-ray micro-computed tomography has gained popularity as the method of choice in the evaluation of bone morphology and microarchitecture, *ex vivo*. While a *few micrometres* thick histological slice may or may not be representative of a 360° view of peri-implant tissue (Shah et al., 2019), X-ray based tomographic techniques are unreliable for measuring the BIC since voxels comprising the immediate peri-implant bone often must be excluded due to the presence of metal artefacts (Liu et al., 2012, Palmquist et al., 2017).

Increase in RTQ (e.g., as a function of time) can be a result of progressive increase in the mineral content of bone, which would be expected to increase the stiffness of the extracellular matrix (Fratzl et al., 2004). Furthermore, dictated by the progression of contact osteogenesis and distance osteogenesis (Davies, 2007), fusion of the two formation fronts results in a dramatic increase in the mechanical properties of the extracellular matrix (Xie et al., 2021). Therefore, RTQ values may increase independently of the BIC. The relative tissue age (and therefore the degree of mineralisation and mineral maturation) will influence the resistance to shear/torsion offered by the interfacial bone. Specifically with reference to the studies considered here, it is unclear if the material properties of peri-implant bone remain unaltered by antiresorptive agents, especially where RTQ values increase without a corresponding change in morphometric parameters such as BIC (Narai and Nagahata, 2003, Skoglund et al., 2004, Verzola et al., 2015). Precision and sophistication of torque measuring equipment varies from simple handheld torque wrenches to completely digital devices. It is therefore likely that detection of subtle variations in mechanical anchorage are subject to the limitations of the equipment used—more so in animal models where the recorded RTQ values tend to be small (e.g., osteoporotic rats). Moreover, RTQ recording procedures vary from *in situ* measurements immediately upon termination (Verzola et al., 2015, Yi et al., 2017, Lee et al., 2011, Scharff-Baauw et al., 2021) to *ex situ* measurements after a freeze-thaw cycle (Yildiz et al., 2010), formalin fixation (Guimarães et al., 2015) or following an overnight adhesive curing (Lotz et al., 2019). Studies commonly report only the peak RTQ value. However, the work of fracture (i.e., energy uptake until failure) (Skoglund et al., 2004, Wermelin et al., 2008)—an important descriptor in fracture mechanics—is scarcely reported. Information on the applied rotational speed would also be useful to be able to compare RTQ values across studies.

Under tension and compression, the Young's modulus and yield strain of cortical bone are linearly related to the strain rate (Hansen et al., 2008). During RTQ measurement, the bone itself is not expected to experience a significant amount of torsion but while there is shear between the bone surface and the implant surface, any mineralised collagen fibrils mechanically interlocked with the implant surface will be expected to experience a tensile strain. Thus, based on the angular speed, disruption of the bone-implant interface will also occur at different peak torque. Since the torque measurement parameters remain poorly reported, direct comparisons cannot be made between RTQ values from different studies. The amount of bone engaged upon implantation has a large influence on RTQ values, with roughly 50% increase in bicortical vs. monocortical implant placement (Chacon et al., 2006). Unintentional engagement of the opposite cortex during implant placement can lead to increased variation within the experimental group, thereby masking the true effects of the treatment plan.

In certain cases, the experimental design makes it possible to determine if the effect of a given drug is merely transient, i.e., only observed during the early stages of healing, or long-term. BMPs tend to exert a strong influence on BIC and RTQ during the early stages of healing (Bessho et al., 1999, Zhang et al., 2021). However, the difference between the RTQ values of BMP treated and untreated groups diminishes with time. In the context of experimentally-induced osteoporosis, a spontaneous reduction in RTQ is observed not only when implants are placed in established osteoporotic conditions (Narai and Nagahata, 2003), but also when the onset of osteoporosis occurs after the implants osseointegration (Giro et al., 2007).

Important parameters to consider in the experimental setup are dose and dosing intervals. In addition to conservative/low doses, problems may arise with very high doses. This is exemplified by the sharp decrease in RTQ reported for topical application of ∼10 mg alendronate as a gel, which is likely to prolong the duration of exposure to the drug and supresses normal bone metabolism (Guimarães et al., 2015).

Owing to the overall geometry (size and total surface area) of the implant, certain surfaces (such as the machined surface) when tested in small animal models (i.e., rats) generate very low RTQ values. Random errors including misaligned/tilted implant or anatomical variations (e.g., cortical bone thickness) may result in large variances within the obtained data, and consequently, subtle differences between experimental groups are liable to be lost (i.e., a case of a ‘trend’ being reported but without ‘statistical significance’). This can be a problem in experiments using a small number of animals per group, and thereby being underpowered. In SS implants of comparable geometry, the RTQ values obtained at 2 weeks of healing vary from 2.3 N cm (Skoglund et al., 2004) to 6.7 N cm (Wermelin et al., 2008), and are comparable to RTQ values measured for titanium implants at analogous time-points, ranging between 4.3 N cm (Lee et al., 2011) and 6.6 N cm (Verzola et al., 2015). A similar observation can be made at 4 weeks where the mean RTQ value of 5.2 N cm for SS implants (Savvidis et al., 2020) is comparable to the mean value of 6.2 N cm for titanium implants of the same diameter and at the same healing period (Lee et al., 2011). Noticeably lower RTQ value of 1.1 N cm recorded at the same time point could be the result of the soft tissue observed at the bone-implant interface (Skripitz and Aspenberg, 2001).

By improving the underlying bone and/or systemic conditions prior to implant placement, ‘priming’ of bone has led to variable levels of success. Oral administration of ALN in healthy rabbits (Chacon et al., 2006), subcutaneous administration of IBN in OVX rats (Lotz et al., 2019), or subcutaneous administration of ALN in combination with whole-body vibration in OVX rats (Shibamoto et al., 2018) have largely been unsuccessful at demonstrating a beneficial impact. In contrast, subcutaneous administration of hPTH together with whole-body vibration has found to be effective in additive manner (Shibamoto et al., 2018). Delayed alveolar socket healing and altered expression of genes involved in the regulation of bone turnover are noted in spontaneously hypertensive rats (Manrique et al., 2015). As such, the beneficial effects of losartan on osseointegration (Mulinari-Santos et al., 2018), are likely related to homeostatic regulation of the vascular system. Orally administered ASU is shown to have a positive, though limited, impact on RTQ in experimentally-induced arthritis but little or no effect in otherwise healthy rats (de Paula et al., 2018). The detrimental effects of cyclosporin A on peri-implant healing have been explored by beginning cyclosporin A administration prior to implant insertion (Sakakura et al., 2003). In contrast, the effect of rhFGF-4 administration directly at the planned implantation site several weeks before implant placement, followed by creation of a drill hole at the time of insertion, remains to be definitively established (Stenport et al., 2003). It must be noted that studies have not, specifically, made comparisons between *pre-implantation* vs. *at the time of implantation* vs. *post-implantation* drug administration.

In some cases, the primary effects of a given substance tend towards correction of the underlying condition, and with it the healing potential, rather than bolstering osseointegration or peri-implant healing. High blood pressure has been correlated with low bone mineral density and lower serum calcium concentration (Gotoh et al., 2005). With addition of losartan, RTQ values in spontaneously hypertensive rats were improved, but addition of losartan to normotensive animals showed no significant effect (Mulinari-Santos et al., 2018). Another example is of type 2 diabetes mellitus where advanced glycation endproducts are involved in non-enzymatic crosslinking of collagen, thus altering bone material properties and decreasing overall bone strength (Yamamoto and Sugimoto, 2016, Hunt et al., 2019). With the addition of aminoguanidine, an inhibitor of advanced glycation endproducts accumulation, RTQ values in hyperglycaemic rats increased while no effect was recorded in normoglycemic animals (Guimarães et al., 2011).

Increased insulin levels can supress bone resorption, as evident from decreased circulating levels of C-telopeptide of type I collagen, without apparent effects on bone formation, while changes in the levels of both carboxylated and uncarboxylated forms of osteocalcin remained dose and time dependent (Ivaska et al., 2015). In animals where diabetes is induced after implants have been allowed to osseointegrate, insulin administration tends to have a protective effect on RTQ but without a detectable variation in BIC between normoglycaemic and hyperglycaemic (treated or untreated) animals (de Molon et al., 2013). However, the positive effects of insulin on biomechanical anchorage of implants are not consistently reported. Despite establishing and maintaining normoglycemic state in rabbit model, insulin administration failed to improve RTQ values of implants (Margonar et al., 2003).

The protective effects of glucocorticoids on bisphosphonate-induced osteoclast apoptosis are well-documented (Weinstein et al., 2002). However, despite systemic administration of dexamethasone, an extremely high incidence of ONJ is observed with subcutaneously delivered ALN which is in contrast to local release of ZLN (i.e., from the implant surface) where peri-implant bone does not show any evidence of ONJ (Abtahi et al., 2013).

Magnesium depletion affects all phases of skeletal metabolism, with severe Mg deficiency leading to loss of bone mass (Wallach, 1990, Rude et al., 2003, Rude et al., 2005). Certain drugs such as cyclosporin A and cisplatin are well known to cause medication induced hypomagnesemia (Atsmon and Dolev, 2005), which may be an explanation for the detrimental effects of cyclosporin A (Sakakura et al., 2003, Sakakura et al., 2006) and cisplatin (Dantas et al., 2019) on RTQ. Direct effects of hypomagnesemia on RTQ have been reported in rats. If the availability of dietary magnesium drops to ∼10% (i.e., 90% reduction), implants that have previously been allowed to heal for up to 60 days, show significant losses in biomechanical anchorage at 60 days (Del Barrio et al., 2010) and 90 days (Belluci et al., 2011) after initiating a low magnesium diet. Additionally, a 90% reduction in dietary magnesium also leads to significant reduction in the bone mineral density of lumbar vertebrae (Del Barrio et al., 2010). Surprisingly, a 75% reduction in dietary magnesium levels is well tolerated, without a significant impact on RTQ or bone mineral density (Del Barrio et al., 2010).

**Figure 6:**
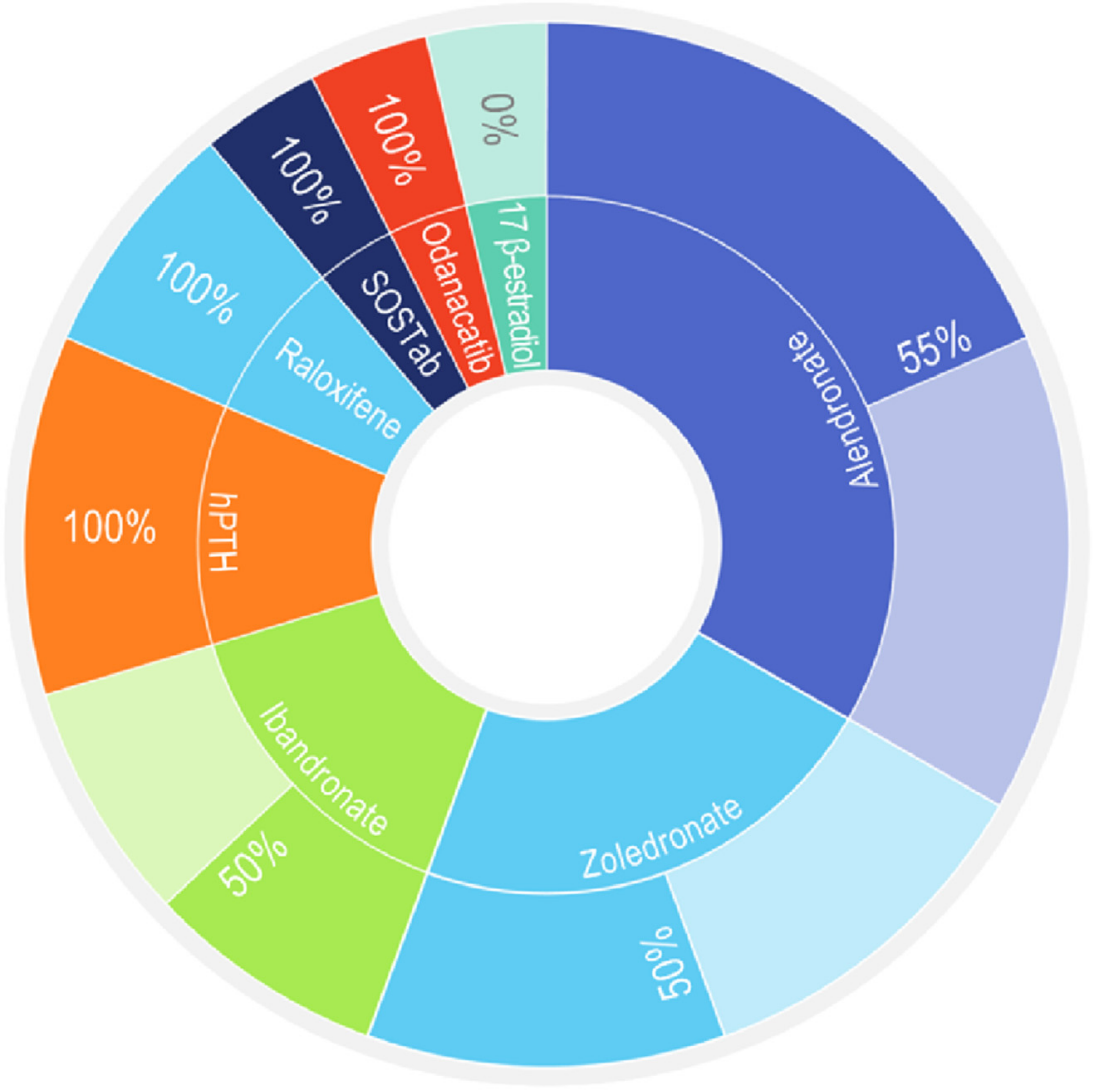
Prevalence of the use of individual antiresorptives included in this review, based on 19 published studies. Four studies have tested at least two antiresorptive agents. The outer ring represents success rates of individual antiresorptives based on the proportion of studies reporting significantly increased RTQ values. One study has used a combination of ibandronate and pamidronate.

Bisphosphonates, raloxifene, oestrogen replacement, and denosumab are known to interfere with bone turnover processes resulting in increased bone density (Recker and Armas, 2011), and their impact on osseointegration warrants further investigation. Of those, BPs have been used with considerable success during last five decades in treatment of osteoporosis, Paget's disease, osteolytic-bone metastases, and cancer-related hypercalcemia (Russell, 2011). They are highly effective inhibitors of osteoclast-mediated bone resorption with high affinity for bone mineral (Fleisch et al., 1969, Fleisch et al., 2002). Inhibition of osteoclast activity is dependent on the molecular structure of the BPs—nitrogen containing BPs being most potent (Rogers et al., 2011). A consistent pattern in the effect of bisphosphonates on the mechanical anchorage of implants could not be recognised here (either in ovariectomised or healthy animals), with 8 out of 16 studies reporting a significant improvement of RTQ values (Figure 6).

It is well established that hPTH has anabolic effects on bone without compromising bone quality (Mosekilde et al., 1991), and it has been shown that hPTH enhances healing in both healthy and ovariectomised rat models (Nozaka et al., 2008). Across the few studies involving hPTH in this review, significant increases in RTQ were recorded in all cases of OVX (Shibamoto et al., 2018) and non-OVX (Skripitz and Aspenberg, 2001) rats, as well as non-OVX rabbits (Corsini et al., 2008).

Reporting on bulk implant material and surface preparation is inconsistent, while drug release profiles for implant surface bound agents (Harmankaya et al., 2013) are infrequently performed and/or reported. In systemic administration, while drug dose is routinely adjusted to animal weight, it remains unclear if the dosage is continually adjusted to accommodate for changes in animal weight during the experimental period.

It must be borne in mind that findings of *in vivo* animal experiments may or may not have direct relevance to the human condition owing to interspecies differences in material composition, bone microstructure, and remodelling patterns (Pearce et al., 2007). Across species, differences in bone mineral density correlate with mechanical properties (Aerssens et al., 1998) such as Young's modulus and strength (Liebschner, 2004). However, physiological parameters such as the average life span and age of skeletal maturity also affect experimental outcomes. In contrast to human bone where circumferential lamellar bone and extensive Haversian remodelling are observed, primary bone predominates in rodents and lagomorphs (Wancket, 2015). In rats, Haversian remodelling is absent while bone modelling continues throughout life and trabecular bone mass is limited. In rabbits, not only is there higher trabecular bone mass, Haversian remodelling is also known to occur (Muschler et al., 2010). Many parallels can be drawn between human bone and canine bone, including Haversian remodelling, age-associated bone loss, and comparable material composition (i.e., organic, inorganic, and water fractions) (Wancket, 2015). Despite the similarities, very few studies are conducted using canine models, which is also evident from the low proportion of such publications (only 1 out of 49) included in this review.

## 7 The way forward

Based on the information gathered in this review, several points urgently warrant further attention. These include: *(i)* characterising how rapidly a given drug is metabolised in a chosen animal species (including *in vivo* models of human disease) and how similar the bioavailability of the same drug is to that in humans; *(ii)* ruling out false negative outcome(s) due to subtherapeutic dosing and/or duration of administration where a therapeutic agent has not been found to influence RTQ; (*iii*) investigating the true severity of impact on bone material properties in humans, particularly in the long-term, where a therapeutic agent has been found to adversely affect RTQ; and (*iv*) determining the short- and long-term consequence(s) of discontinuing bone anabolic/anti-catabolic therapeutic agents that positively impact RTQ, e.g., reduction in mechanical anchorage. It is apparent that many substances have been found to have a positive effect on biomechanical anchorage and these being the most promising should be prioritised in future studies rather than certain all-encompassing miracle cures.

## Declarations

### Author contribution statement

Furqan A. Shah; Conceived and designed the experiments.

Martina Jolic, Sonali Sharma; Performed the experiments.

Martina Jolic, Sonali Sharma, Anders Palmquist, Furqan A. Shah; Analyzed and interpreted the data.

Martina Jolic, Anders Palmquist, Furqan A. Shah; Wrote the paper.

### Funding statement

This work was supported by IngaBritt och Arne Lundbergs Forskningsstiftelse, Stiftelsen Handlanden Hjalmar Svenssons, Adlerbertska Research Foundation, Kungliga Vetenskaps- och Vitterhets-Samhället i Göteborg, and Vetenskapsrådet.

### Data availability statement

Data included in article/supp. material/referenced in article.

### Declaration of interest’s statement

The authors declare no conflict of interest.

### Additional information

No additional information is available for this paper.
